# Comprehensive assessment of adults severely ill with TB referred after triage in Tamil Nadu, India

**DOI:** 10.5588/pha.26.0059

**Published:** 2026-05-18

**Authors:** V. Rajan, H.D. Shewade, A. Frederick, S. Shanmugasundaram, A. Jeyakumar, R. Vijayaprabha, K. Gayathri, K. Rambha, J.M. Melfha, D. Kabir, R. Srinivasan, R. Sabarinathan

**Affiliations:** 1 Division of Health Systems Research, ICMR National Institute of Epidemiology (ICMR-NIE), Chennai, India;; 2 Directorate of Medical and Rural Health Services, Government of Tamil Nadu, Chennai, India;; 3 Division of ICMR School of Public Health, ICMR National Institute of Epidemiology (ICMR-NIE), Chennai, India;; 4 Division of Infectious Disease Epidemiology, ICMR National Institute of Epidemiology (ICMR-NIE), Chennai, India;; 5 Division of Computing and Information Sciences, ICMR National Institute of Epidemiology (ICMR-NIE), Chennai, India.

**Keywords:** tuberculosis, TN-KET, differentiated TB care, Tamil Nadu, COPD, diabetes

## Abstract

Tamil Nadu *Kasanoi Erappila Thittam* (TN-KET) is India’s first state-wide differentiated TB care initiative to reduce TB deaths. Since April 2022, this involved triaging for severe illness at diagnosis and prioritisation of severely ill for inpatient care. In the third year (2024), TN-KET sustained its performance in comprehensive assessment of severely ill during inpatient care, possibly contributing to low deaths in 2024. The information on reasons for severe illness provided will guide strengthening of infrastructure during inpatient care. While therapeutic nutrition and diabetes care is work in progress, sustained efforts are required for managing severe alcohol addiction and chronic lung disease.

Tamil Nadu is a southern Indian state with microbiologically confirmed adult pulmonary TB prevalence of 212 per 100,000 population.^1^ Deaths among notified people with TB mainly occur in the first two months of treatment (early deaths).^2^ To reduce these early deaths, from April 2022 onwards, Tamil Nadu implemented the first state-wide differentiated TB care initiative called Tamil Nadu *Kasanoi Erappila Thittam* (TN-KET, meaning Tamil Nadu TB death-free project in Tamil).^3–5^

All notified adults (≥15 years) with TB (not known to be drug-resistant at diagnosis) were triaged for severe illness at diagnosis using a paper-based triage form. Those with very severe undernutrition (body mass index ≤14 kg/m^2^ or 14.1–16 kg/m^2^ with pedal oedema) or respiratory insufficiency (respiratory rate more than 24 per min or oxygen saturation <94%) or poor performance status (unable to stand without support), defined as ‘triage-positive’ (henceforth, referred to as severely ill), were prioritised for referral, comprehensive assessment (using a single paper-based case record form [CRF]), and inpatient care (using the inpatient care guide).^3–5^ In the CRF, the total score post comprehensive assessment was a composite score (max 32) using 16 indicators. Additionally, there were a total of 22 reasons for severe illness. A paper-based therapeutic nutrition clinical tracking tool was additionally used during inpatient care (stabilisation phase) among severely ill adults with very severe undernutrition. See Figshare (https://doi.org/10.6084/m9.figshare.24564403). After managing their severe illness, the outcome of admission was documented in the CRF and they were discharged for ambulatory care.

Key variables from the paper-based triage form and CRF were captured in Severe TB Web Application (TB *SeWA*).^3–5^ Along with availability of isolation TB beds and therapeutic nutrition, documentation of total scores post comprehensive assessment and the reason for severe illness (transcribed into TB *SeWA* from the CRF) was a surrogate indicator for quality of inpatient care. The TN-KET two-page standard operating procedure, containing links to the paper-based triage tool, the CRF, the inpatient care guide, and details on the monitoring and evaluation framework, can be accessed on Figshare (https://doi.org/10.6084/m9.figshare.24564403).

TN-KET was implemented in all the 30 districts of Tamil Nadu (except Chennai, capital city). Chennai being the capital city, it has clinical and diagnostic capacity to comprehensively assess adults with TB at diagnosis (without the need to triage and prioritise ‘triage-positive’ severely ill for comprehensive assessment).

During October to December 2022, though 90% severely ill were documented to have undergone comprehensive assessment (‘yes’ in TB *SeWA*), total score was documented in 39% only.^6^ Field level exploration revealed that the CRF was used only in 23% of severely ill.^6^ Guided by these findings, the districts were reorientated by the state TB cell, resulting in improvement in availability of total scores in 2023 (70%–80%).^6^

## RESULTS

We wanted to assess if the documentation of total score post comprehensive assessment was sustained or improved during the third year TN-KET (2024). Additionally, TB SeWA data on reasons for severe illness could guide the services that needed to be scaled up in the nodal inpatient care facilities.

**FIGURE 1.**
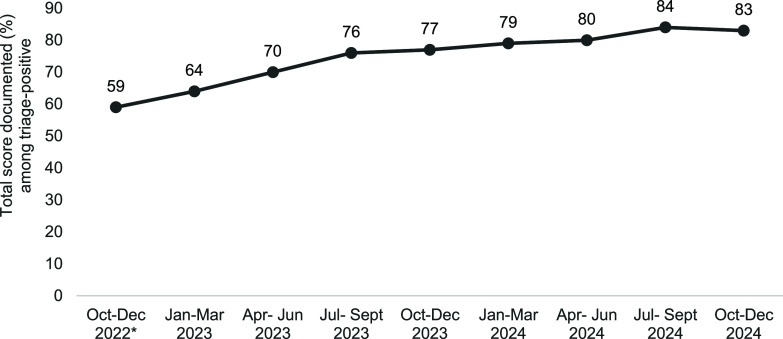
Quarter-wise trend of documentation of total score post comprehensive assessment in TB *SeWA* among triage-positive (severely ill at diagnosis) under TN-KET, Tamil Nadu, India. TB *SeWA* = Severe TB Web Application; TN-KET = Tamil Nadu *Kasanoi Erappila Thittam*; CRF = case record form; *TN-KET started in April 2022; however, case record form for comprehensive assessment was introduced in August 2022, and hence this indicator is available starting October to December 2022 diagnosis cohort.

**FIGURE 2.**
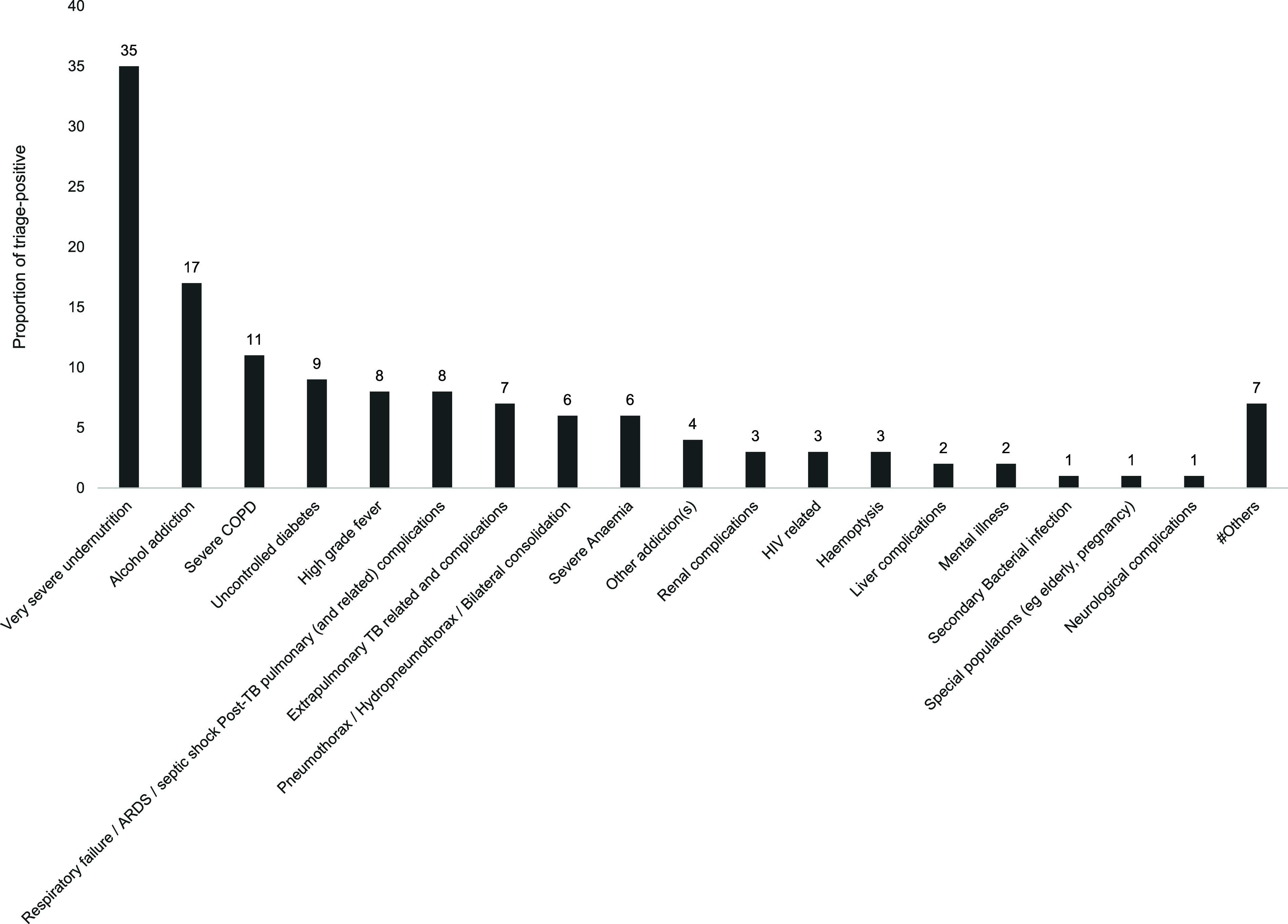
Distribution of reasons for severe illness among triage-positive for whom reason for severe illness was documented* in TB *SeWA* under TN-KET, Tamil Nadu, India, 2024 (N = 5,569). TB *SeWA* = Severe TB Web Application; TN-KET = Tamil Nadu *Kasanoi Erappila Thittam*; COPD = chronic obstructive pulmonary disease; ARDS = acute respiratory distress syndrome. *Among the 6,745 severely ill, the reasons for severe illness were documented in 5,569 (83%). Multiple reasons can be present for one individual. ^#^Others include deep venous thrombosis and pulmonary embolism, immune reconstitution inflammatory syndrome, complications to anti-TB treatment (adverse drug reaction), post-COVID-19 sequelae or adult with TB is sick but not classified into above categories.

Among the 53,793 public notified adults with TB in Tamil Nadu in 2024, 52,985 (99%) were triaged, of which 6,745 (13%) were severely ill. Among the 6,745 severely ill, 6,313 (94%) were comprehensively assessed and 5,483 (81%) had documented total scores post comprehensive assessment (see Figure 1). Among the 6,745 severely ill, the reasons for severe illness were documented in 5,569 (83%). The common reasons for severe illness were very severe undernutrition (35%), alcohol addiction (17%), severe chronic obstructive pulmonary disease (COPD) (11%), and uncontrolled diabetes (9%) (see Figure 2). One, two, and three reasons were documented in 46%, 12%, and 25%, respectively.

## DISCUSSION

In the third year of TN-KET, we observed sustained high levels of documentation of comprehensive assessment among severely ill adults with TB in routine program settings. The information on reasons for severe illness provided will guide strengthening of infrastructure during inpatient care. However, missing data (17%) could bias the distribution of reasons for severe illness. We have not compared the baseline characteristics of the two populations that had the reasons documented and that had not. This is a limitation.

More than four fifths of severely ill adults with TB had documented total score and reasons for severe illness, indicating that practices introduced during earlier phases of TN-KET have become embedded within routine inpatient care. This sustained performance suggests consolidation of system-level capacity for managing severe TB illness rather than a transient improvement following program reorientation^7^ (see Figure 1).

Very severe undernutrition and alcohol addiction appear to be the two most common reasons for severe illness. This emphasises the need for therapeutic nutrition during inpatient care; recommended by India’s Central TB Division and prioritised in TN-KET.^7^ In National Capital Territory of Delhi (2024), TN-KET was replicated and we were able to systematically document the improvement in performance status among very severely undernourished adults with TB following therapeutic nutrition during inpatient care.^8^ In 2024, around ≈20 districts of Tamil Nadu provided therapeutic nutrition in their nodal inpatient care facilities. While these ≈20 districts should sustain this, the other districts should initiate the provision of therapeutic nutrition in their district teaching hospital (and if possible, in the district headquarter hospital).

For management of alcohol addiction, effective linkage to mental health services (psychiatry department) during inpatient care and appropriate follow up post discharge should be strengthened. Since October 2023, TB *SeWA* had the option to follow up severely ill adults with TB post-discharge at 1 month and 2 months using the same triage tool. In 2024, ≈30% severely ill were followed up at 2 months. Routinely within the program, all those on treatment are followed up at 2 months in the nearest public health facility for microbiological conversion. This opportunity should be leveraged for follow-up triaging. This aspect of TN-KET needs strengthening.

Chronic conditions, like COPD and diabetes, also represent a significant burden among severely ill people, reinforcing the significance of long-term disease management strategies. For diabetes, with the aim of improving the glycaemic control among people with TB, in 2025, a TB-DM module has been added to TB *SeWA*. Capillary fasting blood glucose and insulin use is documented (at diagnosis, 2 months TB treatment, successful completion of TB treatment) for all public notified people with TB who have diabetes. Insulin is being prioritised for those with capillary fasting blood glucose >250 mg/dL.

WHO in their consolidated guidelines on TB and comorbidities recommends comprehensive response to TB and comorbidities.^9^ Comprehensive assessment and quality inpatient care among severely ill is a step in this direction. When compared to TB death rate of 7% for 2021 cohort and 6% for 2019 cohort (pre-COVID-19), the state has documented 30% TB death rate reduction in 2024. While improvements in the management of comorbidities is work in progress, the improved quality of comprehensive assessment and inpatient care likely contributed to reduced deaths in 2024.
